# A borderline personality disorder diagnosis may improve clinical psychology trainees' treatment beliefs

**DOI:** 10.1111/bjc.70076

**Published:** 2026-06-12

**Authors:** Katie E. Hastings, Adam Hahn

**Affiliations:** ^1^ Avon and Wiltshire Mental Health Partnership NHS Trust Swindon UK; ^2^ University of Bath Bath UK

**Keywords:** borderline personality disorder, clinical associate in applied psychology, clinical psychology doctorate, clinical psychology trainees, diagnostic label, old age, stigma, wider psychological workforce

## Abstract

**Background:**

Contentious debate persists over whether the stigma of borderline personality disorder (BPD) stems from the diagnostic label itself or the behaviours commonly associated with it. At the same time, it remains unclear whether old age identities exacerbate such stigma, raising concerns surrounding the quality of care experienced by the burgeoning population of older adults—both diagnosed and undiagnosed—presenting to mental health services with BPD symptoms.

**Method:**

Using a 2 × 2 between‐subjects experimental design, participants were randomized equally to one of four conditions varying by age (27 vs. 70 years) and diagnostic label (BPD vs. no diagnosis). The study used two‐way independent‐measures ANOVAs to examine 242 clinical psychology trainees' treatment beliefs, as well as their cognitive, emotional and behavioural reactions to a vignette character presenting with BPD symptoms.

**Results:**

Contrary to expectations, participants reported more favourable treatment beliefs about patients with a BPD diagnosis relative to undiagnosed patients with identical BPD symptoms, as indicated by more positive beliefs about their therapeutic relationships, and more conviction in their treatability. Additionally, participants anticipated that they would exhibit more infantilising behaviours (i.e., child‐like speech patterns) toward older adult patients than toward younger patients, independent of diagnostic label.

**Conclusions:**

A BPD diagnostic label can improve clinical psychology trainees' expectations about the treatability of affected individuals and may have a positive influence on beliefs about the therapeutic relationship with younger adult patients in particular. Thus, the label may now be less stigmatising among current clinical psychology trainees compared to previous generations.

**Practitioner points**

- Patients with a BPD diagnosis may benefit from more positive treatment expectations than their undiagnosed counterparts.
- A patient's old age identity can have an inappropriately negative impact on clinical psychology trainees' anticipated use of infantilising behaviours.
- Clinicians should therefore be cautious in withholding the BPD diagnosis solely due to concerns around how other mental health providers might respond.
- Clinical psychology doctorate and clinical associate psychology courses might consider including reflective practices surrounding one's reactions to old age.

A prominent debate in discussions on borderline personality disorder (BPD) revolves around its status as one of the most stigmatized psychiatric conditions in clinical practice (Ahmed et al., 2021; Ferguson, 2016; Hazell et al., 2022; Markham & Trower, 2003; Masland et al., 2022; Sheehan et al., 2016), with mental health professionals' descriptions of BPD patients as dangerous and manipulative showing no signs of abating (Deans & Meocevic, 2006; Gallop et al., 1989; Gallop & Wynn, 1987; Nehls, 1998; Papathanasiou & Stylianidis, 2022; Servais & Saunders, 2007; Woollaston & Hixenbaugh, 2008).

Early publications listed characteristics of BPD—such as emotional instability and self‐harming behaviours—as reasons for the disproportionate stigma plaguing affected individuals (Aviram et al., 2006; Dickens et al., 2016; Fraser & Gallop, 1993; Kelly & May, 1982; Maltsberger & Buie, 1974; Nehls, 1999; Stotland, 1998). Later writers have contested this, citing findings that professionals express more social rejection and dangerous stereotypes toward patients with BPD than those with schizophrenia and major depressive disorder (Fraser & Gallop, 1993; Markham, 2003; Markham & Trower, 2003), even when behaviours associated with these conditions are often comparable (Koekkoek et al., 2006; Sulzer, 2015).

Unique to this disorder, though, is the interpersonal dysfunction, which is prominent in the clinical portrait of BPD across the lifespan (Videler et al., 2019; Winsper, 2021). Characterized by frantic efforts to avoid abandonment and a tendency to oscillate between idealising and devaluing others (American Psychiatric Association, 2013), these interpersonal difficulties can put affected individuals at odds with others, including professionals (Ahmed et al., 2021). For example, McGrath and Dowling (2012) describe qualitative findings from a sample of 17 psychiatric nurses indicating that conceptualisations of patients with BPD as manipulative were often precipitated by experiences where a patient was initially pleasant but quickly became aggressive. Such findings may reflect that, given the intense emotional states often expressed by affected individuals within interpersonal contexts (Miskewicz et al., 2015; Sadikaj et al., 2010), clinicians may experience patients' projections of distress as personal attacks (Fraser & Gallop, 1993) and develop fixed negative beliefs about BPD to protect themselves from self‐blame (Aviram et al., 2006; Fraser & Gallop, 1993). It is perhaps unsurprising, then, that scholars have long attributed BPD stigma to the actual behaviours seen among this patient group, which—if this notion is to be believed—would indicate that individuals exhibiting BPD traits are likely to experience stigmatising reactions from clinicians, even in the absence of a formal diagnosis.

However, in contrast to professionals' subjective reports that their stigmatising responses stem from specific BPD symptoms, research has also documented that clinicians continue to espouse stigmatising views about the disorder that cannot be linked to actual patient presentations. For example, in a sample of 22 mental health professionals, most reported that their colleagues associated the BPD label with “untreatability” and subsequently denied this population care (Sulzer, 2015), despite widely recognized and carefully controlled trials showing that the symptoms may be as treatable as those of Major Depressive Disorder (Gunderson et al., 2011).

One common explanation for this discrepancy between professional narratives and patient presentations is that the label itself may acquire misplaced characteristics in the minds of clinicians, shaped by treatment biases that have burdened the diagnosis since its conception as a colloquialism for ‘untreatable neurotics’ (Al‐Alem & Omar, 2008; Gunderson, 2009; Knight, 1953; Stern, 1938; Stone, 1977). In line with this possibility, Lam et al. (2016) presented 265 mental health professionals with a video depicting panic disorder, which varied by the inclusion of a behavioural description consistent with BPD or a BPD diagnostic label. The authors found that the BPD label was associated with more negative ratings of the patient's problems and prognosis than a behavioural description of BPD alone, suggesting the diagnostic label, rather than the behaviour it denotes, activates negative treatment beliefs about affected individuals.

There is also reason to believe that clinicians' stigmatising reactions to BPD patients may be moderated by patient age, with the prevailing double stigma hypothesis asserting that the stigma attached to old age and mental illness is additive (Brown & Adeagbo, 2021; Helmes & Gee, 2003; Lee et al., 2003; Thomas & Shute, 2006). If this theory holds true, the increasing number of older adults entering mental health facilities with a BPD diagnosis (Beatson et al., 2016) may face even greater stigma from professionals than separate considerations might indicate.

Although no research to date has directly examined the double stigma hypothesis in the context of BPD, some evidence exists to support this claim more generally. For instance, Kessler and Blachetta (2018) found that trainee psychotherapists receiving an 86‐year‐old reported more negative predictions about the patient's prognosis compared to those receiving a 56‐year‐old, despite identical symptoms of depression. Such negative treatment beliefs may be rooted in trainees' assumptions that old age is characterized by apathy and reduced psychological mindedness (Lee et al., 2003). This supports the idea that, by virtue of their stigmatized age, clinicians may perceive older adults with BPD as less able to improve with treatment than their younger counterparts. However, as research on this topic is limited and has yet to be explored for BPD, this relationship remains unclear.

To complicate matters further, professionals are less likely to ascribe negative emotional reactions and dangerous stereotypes to older adults (Werner & Segel‐Karpas, 2019). The belief that an individual is dangerous has been found to arise from perceived physical strength (Harris & Miller, 2000). Consequently, stereotypes portraying older adults as weak (Hummert et al., 1994) may attenuate perceptions of danger commonly associated with BPD and, in turn, lessen fear or social distance reactions (Ellison et al., 2015). This suggests that, rather than simply being additive, the magnitude of stigma elicited by older adults with BPD might depend on the factor of each stigma component examined.

Taken together, existing evidence indicates that older adults with BPD may face stigmatising reactions from mental health providers by virtue of their age and diagnosis in isolation. At the same time, the many effective evidence‐based treatment modalities currently available for BPD (Bateman & Fonagy, 2009; Clarkin, 2007; Doering et al., 2010; Linehan, 1991; Linehan et al., 2006) might have attenuated clinical psychology trainees' negative reactions to the label. That is, BPD symptoms could be perceived as more manageable in the presence of a diagnostic label that now conveys positive treatment outcomes. Further, with suggestions of complex old age and mental health identity interactions, the examination of clinical psychology trainees' reactions to older adults with BPD represents an important area of research.

Hence, the present study aims to address these gaps in the literature: Are older adult patients with a BPD diagnosis rated more negatively on measures of treatment beliefs, cognitive, emotional and behavioural reactions compared with younger and undiagnosed patients? To this end, the present study measured Clinical Psychology Doctorate (ClinPsyD) and Clinical Associate Psychology (CAP) trainees' treatment beliefs, cognitive, emotional and behavioural reactions to a vignette character with BPD symptoms, which was experimentally made to vary by age (young adults = 27 years; older adults = 70 years) and diagnostic label (BPD diagnosed; undiagnosed).

Based on extant literature (Lam et al., 2015, 2016; Sheehan et al., 2016), it was hypothesized that participants would report more stigmatising responses to patients with a BPD diagnosis compared with undiagnosed patients and that these effects would be moderated by patient age (Kessler & Blachetta, 2018). Specifically, the stigma of a BPD diagnosis would be more pronounced in older compared with younger patients.

## METHOD

### Open Science practices

The study was preregistered at https://aspredicted.org/2xb2c.pdf.^1^ All measures and exclusions are reported transparently, with data, analyses and materials freely available on the Open Science Framework at https://osf.io/mf3uh/?view_only=526ab0f34bcf401e8a47d18d69e6eacc.

### Participants

A total of 529 UK clinical psychology trainees were recruited between October 2023 and January 2024 via opportunity sampling – directly messaging ClinPsyD and CAP trainees on LinkedIn and emailing Heads of Departments across 29 UK universities. Using G*Power (Faul et al., 2007), it was determined that 210 participants were required to detect the effect found in a study with similar stimuli (Masland & Null, 2022; Cohens *f* = .182) at *p =* .05 and 80% power in a 2 × 2 ANOVA. The study and all associated procedures received ethical approval from the University of Bath's Psychology Ethics Committee (UGM 23–094).

### Design and experimental manipulations

Participants were randomly assigned to one of four background information conditions by the survey platform, QuestionPro. The four conditions described a patient in a referral letter as either: a 27‐year‐old with BPD, a 70‐year‐old with BPD, an undiagnosed 70‐year‐old or an undiagnosed 27‐year‐old (control). This resulted in a 2 (diagnostic label: BPD diagnosed; undiagnosed) × 2 (age: young adults = 27 years; older adults = 70 years) between‐subjects vignette design.

All conditions were accompanied by the same fictitious patient referral letter, which detailed adverse childhood experiences and symptoms consistent with the clinical portrait of BPD (see Figure 1). Symptom depiction was based on research describing age‐neutral manifestations of BPD (see Trappler & Backfield, 2001; Videler et al., 2019). Specifically, depictions of self‐harm included a “refus[al] to take medication” and “skin‐picking” rather than cutting, as the latter is rarely observed among older patients with BPD (Beatson et al., 2016). Other DSV‐IV BPD criteria included frantic efforts to avoid real or imagined abandonment; unstable and intense interpersonal relationships; recurrent suicidal behaviour or threats; affective instability and chronic emptiness (Biskin & Paris, 2012). For instance, details pertaining to unstable interpersonal relationships and self‐harm included: “Experiencing disappointment and anger with her friend, Ms B. became increasingly distressed and ruminated about committing suicide”. The vignette contained no features suggestive of cognitive impairment or dementia‐related behavioural change, and the patient's difficulties were explicitly characterized as longstanding since adolescence, precluding misinterpretation as a later‐life dementia. A Personality Disorder Specialist Practitioner and a Clinical Geropsychologist concurred that the vignette presented symptomology consistent with their clinical experience and fulfilled appropriate DSM‐IV BPD criteria.

**FIGURE 1:**
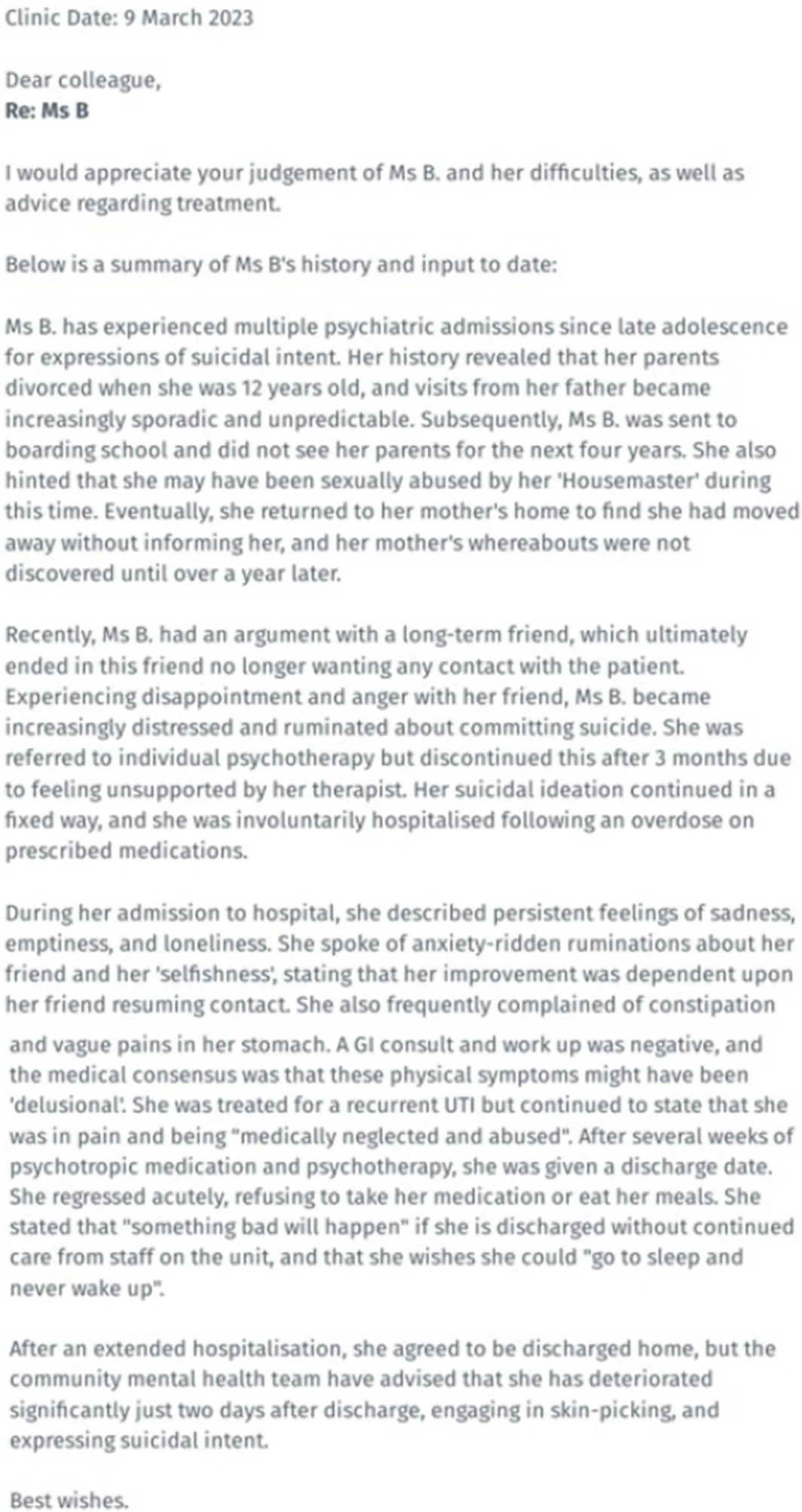
Fictitious patient referral letter.

### Measures

The pre‐registered outcome variables were intended to capture the four distinct components of stigma identified in extant literature (Corrigan, 2000; McKenzie et al., 2022). This included negative treatment beliefs; emotional reactions (i.e., negative emotions, lack of empathy); cognitive reactions (i.e., old age stereotypes, manipulative, dangerous, dependent) and behavioural responses (i.e., social distancing, emotional distancing, infantilising behaviour), which were assessed with one factor each.

Items were scored on 7‐point Likert scales, and all final scores indicate average agreement with these items (1 = *strongly disagree* to 7 = *strongly agree*), with higher mean scores denoting greater levels of stigma. Final internal consistency values (Cronbach's *α*) for each subscale are reported in Table 1. For all remaining scales beyond Treatment Beliefs, values were interpreted against criteria suggested by Nunnally and Bernstein (1994). For *α* values less than 0.6, we deleted items with item‐total correlations < 0.05 (Tavakol & Dennick, 2011).

**TABLE 1:** Cronbach's α scores for each subscale.

Subscales	Cronbach's *α*
Therapeutic alliance	.76
Treatment attendance	.62
Treatment appropriateness	.62
Negative emotions	.77
Empathy feelings	.02*
Manipulative	.68
Dangerous	.76
Dependent	.71
Old age stereotypes	.72**
Social distancing	.85
Emotional distancing	.62
Infantilising behaviour	.75

*Note*: All items can be found in the Open Science Framework.

* All item‐total correlations approached zero, so the empathy feelings subscale was removed.

** Cronbach's α for the revised old age stereotypes scales, item 1 had an item‐total correlation of .002 and was deleted.

#### Treatment beliefs

The Treatment Belief Questionnaire (TBQ) is a 12‐item self‐report measure assessing stigmatising beliefs about a patient's prognosis and engagement with treatment. Items were adapted from Lam et al.'s (2016) Clinical Assessment Questionnaire, which was previously found (Lam et al., 2005) to have good psychometric properties, including excellent test–retest reliability (*r* = .94–.89). An example of an adapted item is: “Ms B would drop out from therapy”. Fifteen original items from Lam et al.'s (2016) questionnaire, relating to perceived likelihood of harm and dependency, were excluded to avoid overlap with cognitive response measures. Pharmacotherapy‐related items fall outside clinical psychology training competencies and so were not considered appropriate in the present sample. Additionally, as the original measure could not capture how patients are stigmatized differently for their age, 4 items concerning old age treatment bias were adapted from James and Haley (1995). For example, “Ms B's presenting complaint may be related to an organic mental disorder”.

However, while treatment belief items were selected from measures with previous evidence of validity, their factor structure was not replicated in the construction of the TBQ. To extract the underlying factorial structure (Tavakol & Wetzel, 2020), twelve treatment belief items were subjected to non‐preregistered exploratory factor analysis with principal axis factoring. Three factors were re‐rotated using direct oblimin rotation and interpreted against criteria suggested by Stevens (2002), i.e., only retaining items with factor loading >0.4. This resulting 3‐factor, 8‐item solution, explaining 47.01% of the total variance, is depicted in Table 2.

**TABLE 2:** Exploratory Factor Analysis of the Treatment Belief Items with Unique Loadings Above 0.4.

Item	Therapeutic Alliance	Treatment attendance	Treatment appropriateness
I would find it easy to establish and maintain a rapport with Ms. B.	.829		
Ms B. could be easily engaged during therapeutic sessions.	.627		
Ms B. would be able to develop an adequate therapeutic relationship with you.	.503		
Ms B. would drop out of therapy.		.680	
Ms B. would miss a lot of therapeutic sessions.		.468	
Ms B.'s condition is treatable.			.716
Ms B. would be an appropriate candidate for psychological therapy.			.488
Ms B.'s interpersonal relationships would improve following treatment.			.554

The first grouping of items suggested a latent variable related to beliefs about the therapeutic alliance. This was based on characterisations of the therapeutic alliance as the therapist's and patient's mutual engagement in therapy (Stubbe, 2018), as well as their collaboration in developing a positive rapport (Ardito & Rabellino, 2011). The second grouping of items suggested a latent variable related to beliefs about treatment attendance (e.g., Bodner et al., 2015), while the final grouping suggested a latent variable related to treatment appropriateness (i.e., whether psychological intervention can treat the condition; Sidani et al., 2009). Thus, these three scales were included as non‐preregistered outcome variables.

#### Emotional reactions

The Emotional Reactions Questionnaire (ERQ) is a 12‐item self‐report measure assessing emotional reactions to a patient. This was adapted from Bodner et al.'s (2011a, 2011b) Borderline patients–Emotional Attitudes inventory, which was found (Bodner et al., 2011a, 2011b) to have good psychometric properties, including good internal consistency (*α* = .60–.84). The first factor included 7 items concerning negative emotions such as anger, impatience and helplessness (e.g., “I would feel angry when Ms B threatened to commit suicide”). The second factor included 5 items measuring empathy feelings (e.g., “I would like to relieve Ms B's suffering”). Bodner et al.'s original ‘experienced treatment difficulties’ factor was excluded to avoid overlap with the TBQ.

#### Cognitive reactions

The ‘Cognitive Reactions Questionnaire’ (CRQ) is a 16‐item self‐report measure assessing participants' endorsement of negative stereotypes about the patient. This was adapted from the Personal Attributes Scale (Angermeyer et al., 2004), covering two factors of mental illness stereotype which have been found (Ellison et al., 2015) to have good psychometric properties, including good internal consistency (*α* = .77 for dangerousness, *α* = .69 for dependency). The first included 4 items concerning perceived dangerousness (e.g., “… psychiatric hospitals should protect the public from people like Ms B”), and the second included 4 items related to perceived dependency (e.g., “patients like Ms B are often needy”). The original measure omitted important BPD and old age stereotypes, so a third factor of ‘manipulativeness’ was included, using 4 items adapted from Bodner et al. (2011a, 2011b). For example, “psychotic manifestations among patients like Ms B are in fact malingering”. The fourth factor concerned ‘old age stereotypes’ which suggest that older adults are lonely, weak, boring and senile, including 4 items developed from Ben‐Harush et al.'s (2016) study (e.g., “patients like Ms B. are often boring”).

#### Behavioural responses

The ‘Behavioural Responses Questionnaire’ (BRQ) is a 16‐item self‐report measure assessing participants' anticipated discrimination against the patient. The first factor included 4 items adapted from Scior and Furnham (2011), which covered social distancing (i.e., willingness to have contact with the patient in situations of increasing intimacy, e.g., “I would not want to move next door to Ms B”). This was previously found (Ellison et al., 2015) to have excellent internal consistency (*α* = .91). This original measure omitted important behavioural reactions to BPD and older adults. Thus, a second factor of ‘emotional distancing’ was included (i.e., emotionally withdrawing from patients through a detached or overly clinical stance), using 4 items constructed from Steinert et al.'s (2019) study (e.g., “I tune out to protect myself…”). The third factor concerned ‘infantilising behaviour’ (i.e., child‐like speech), including 6 items constructed from Noriega et al.'s (2023) study (e.g., “I find myself speaking in a higher pitch…”).

### Procedure

Participants accessed the study through the online software (QuestionPro), where they were initially presented with an information sheet explaining that the study concerned clinical psychology trainees' judgements of a patient and their prediction of treatment outcomes. After completing informed consent, QuestionPro randomly assigned participants in equal proportions to read one of four background information conditions, which varied by age and diagnosis. This was accompanied by a fictitious referral letter describing the patient's presentation, which was identical across conditions to assess whether stigma was a function of the presence of the diagnostic label alone. Participants were then asked to select the age and historical diagnosis of their assigned patient to check that the manipulation had worked. Participants were instructed to indicate the extent to which they agreed with 56 statements comprising the TBQ, ERQ, CRQ and BRQ. Finally, participants answered demographic questions concerning their gender, age, ethnicity and training course. Upon completion, participants were debriefed. No identifying information was collected for any participant, ensuring data was completely anonymous.

### Statistical analysis

Eleven two‐way independent‐measures ANOVAs were run to examine potential interaction effects between the between‐subjects factors of age (young adults = 27 years; older adults = 70 years) and diagnostic label (BPD diagnosed; undiagnosed) on each continuous outcome variable. As preregistered, significant effects were followed up with simple effect analyses to determine the precise patterns of effects with least significant difference calculations. For each outcome, we report estimated marginal means (M) per condition (on 1–7 scales), their associated standard errors (SE) and 95% confidence intervals (CI) from the ANOVA analyses.

## RESULTS

### Participant exclusions

As preregistered, 230 of the 529 initially recruited participants were excluded for incomplete survey responses, 51 for failing at least one of two manipulation check items and six for failing to confirm whether they were on the ClinPsyD or CAP course. The final sample included 242 participants (see Table 3; demographic characteristics). An unequal sample of ClinPsyD (*N* = 206) and CAP (*N* = 36) trainees was recruited, undermining reliable group comparisons, so the pre‐registered exploratory objective of comparing attitudes by course was not examined. The proportion of participants in each condition did not significantly differ by gender (*χ* ^2^(3, *N* = 239) = 3.06, *p* = .38), ethnicity (*χ* ^2^(3, *N* = 241) = 0.49, *p* = .92) or age (*F*(1, 237) = 2.270, *p* = .081).

**TABLE 3:** Participant demographic characteristics (ethnicity, age and gender) across experimental conditions.

	Condition				Total
	Diagnosed, young	Diagnosed, older	Undiagnosed, young	Undiagnosed, older	
Mean age (Years)	28.41	27.04	29.01	28.52	28.35
Female	57	38	60	51	206
Male, Non‐binary, Gender fluid^a^	11	5	5	10	31
White	55	37	51	49	192
Black	3	3	4	5	15
Asian	5	2	4	4	15
Multiracial	5	2	5	2	14
Other	0	1	2	2	5

^a^ Participants identifying as male, non‐binary or gender fluid were grouped in this table due to small numbers within each category, which limited the feasibility of conducting meaningful statistical comparisons. Combining these categories allowed for sufficient statistical power while maintaining inclusion of all participants.

### Treatment beliefs

#### Therapeutic Alliance

Results showed that neither the patient's age, *F*(1, 238) = 0.26, *p* = .608, *η*_*p*_ 2 = 0.001, nor the patient's diagnostic label, *F*(1, 238) = 0.31, *p* = .578, *η*_*p*_^*2*^ = 0.001, showed significant effects on participants' predictions about the therapeutic alliance. However, there was a significant interaction between patient age and diagnosis, *F*(1, 238) = 4.42, *p* = .037, *η*_*p*_^2^ = 0.018. Participants who were assigned a young patient made more negative predictions about the therapeutic alliance in response to the undiagnosed patient (M = 3.70, SE = .112, 95% CI [3.480, 3.923]) than the diagnosed BPD patient (M = 3.38, SE = .112, 95% CI [3.162, 3.602]), *F*(1, 238) = 4.05, *p* = .045, *η*_*p*_^*2*^ = 0.017 (see Figure 2, Panel A). However, for participants who were assigned an older adult patient, predictions about the therapeutic alliance did not significantly differ for the patient with a BPD label (M = 3.70, SE = .137, 95% CI [3.426, 3.967]) and the undiagnosed patient (M = 3.51, SE = .117, 95% CI [3.280, 3.741]), *F*(1, 238) = 1.059, *p* = .305, *η*_*p*_^*2*^ = 0.004.

**FIGURE 2:**
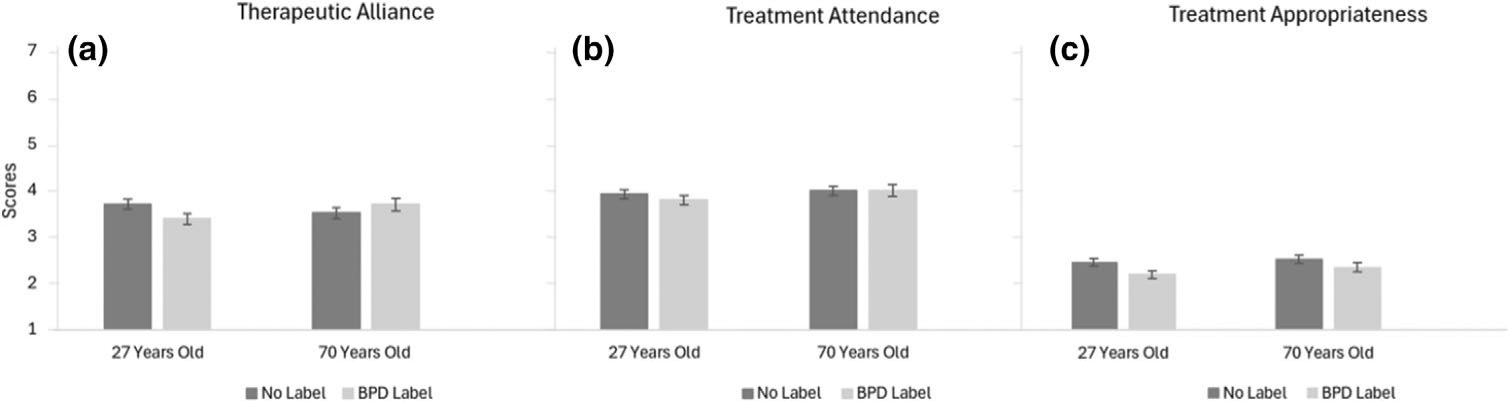
Clinical Psychology Trainees' Scores on the Therapeutic Alliance, Therapeutic Attendance and Treatment Appropriateness Subscales by age (young adults = 27 years; older adults = 70 years) and diagnostic label (BPD diagnosed; undiagnosed). Figures 1, 2, 3, 4 present Clinical Psychology Trainees' scores on 1–7 scales, with higher scores indicating more negative expectations. Error bars represent standard errors.

#### Treatment attendance

Results showed that neither the patient's age nor the patient's diagnostic label nor their interaction showed significant effects on participants' predictions about the patient's treatment attendance, with all *F*s < 1.49 and all *p*s > .223 (see Figure 2, Panel B).

#### Treatment appropriateness

Results showed a significant main effect of BPD, *F*(1, 238) = 6.04, *p* = .015, *η*_*p*_^*2*^ = 0.025. As shown in Figure 2 (see Panel C), this indicated that participants had more positive predictions about treating patients with a BPD diagnosis (M = 2.28, SE = .066, 95% CI [2.379, 2.618]) than undiagnosed patients (M = 2.50, SE = .061, 95% CI [2.148, 2.409]). A non‐significant main effect of age, *F*(1, 238) = 1.60, *p* = .207, *η*_*p*_^*2*^ = 0.007, showed that there was no significant difference in beliefs about treating older adult patients compared to younger patients. Additionally, there was no significant interaction, *F*(1, 238) = .21, *p* = .65, *η*_*p*_^*2*^ = 0.001, revealing that independent of the patient's age, participants made more positive predictions about treating patients with a BPD diagnosis compared with undiagnosed patients.

### Emotional and cognitive reactions

Results showed that neither the patient's age, nor the patient's diagnostic label, nor their interaction showed significant effects on participants' negative emotional reactions, or endorsement of manipulative, dependent, old age and dangerous stereotypes, with all *F*s < 1.19, and all *p*s > .130 (see Figure 3 and Figure 4, Panels A–D).

**FIGURE 3:**
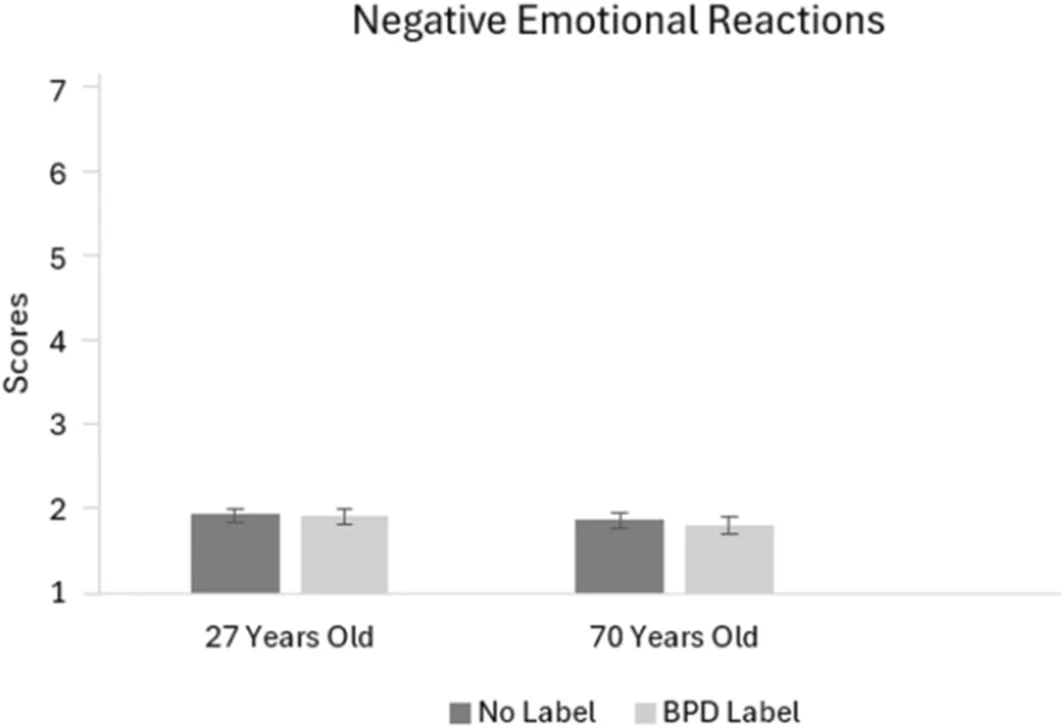
Clinical Psychology Trainees' Negative Emotional Reaction Scores by age (young adults = 27 years; older adults = 70 years) and diagnostic label (BPD diagnosed; undiagnosed).

**FIGURE 4:**
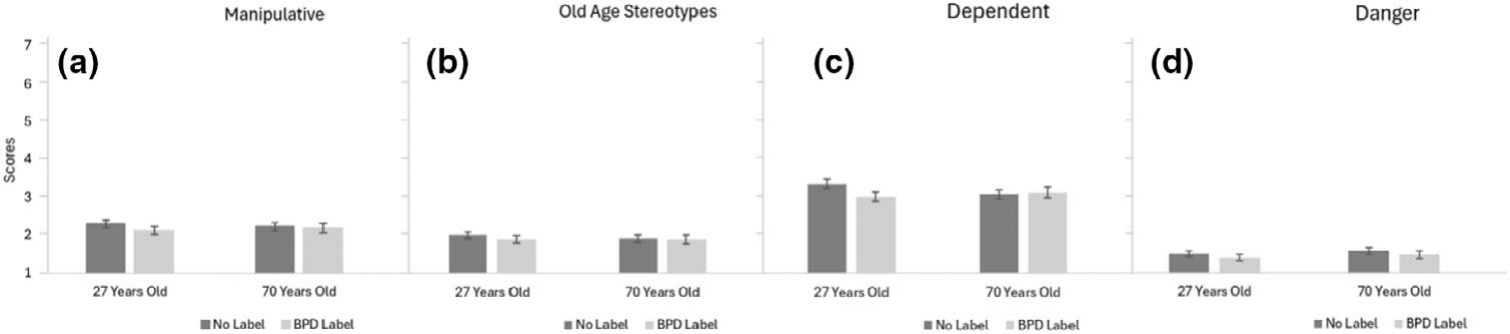
Clinical Psychology Trainees' Scores on the Manipulative, Old Age Stereotypes, Dependent and Danger Subscales by age (young adults = 27 years; older adults = 70 years) and diagnostic label (BPD diagnosed; undiagnosed).

### Behavioural responses

Results showed that neither the patient's age, diagnosis, nor their interaction had a significant effect on participants' anticipated social or emotional distancing, with all *F*s <1.04 and all *ps* > .309 (see Figure 5, Panels A–B).

**FIGURE 5:**
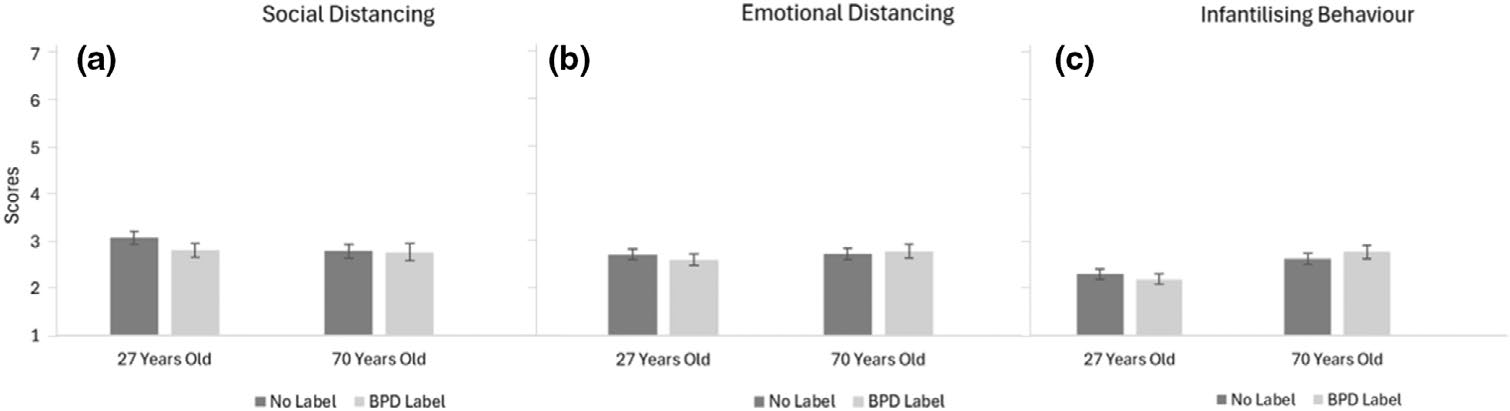
Clinical Psychology Trainees' Scores on the Social Distancing, Emotional Distancing and Infantilising Behaviour Subscales by age (young adults = 27 years; older adults = 70 years) and diagnostic label (BPD diagnosed; undiagnosed).

#### Infantilising Behavioural reactions

As predicted, results showed a significant main effect of age, *F*(1, 238) = 14.10, *p* < .001, *η*_*p*_^*2*^ = 0.056. As shown in Figure 5 (see Panel C), this indicated that participants anticipated that they would use more infantilising behaviours toward older patients (M = 2.72, SE = .091, 95% CI [2.537, 2.895]) than younger patients (M = 2.26, SE = .080, CI [2.103, 2.418]). A non‐significant main effect of BPD, *F*(1,238) = 0.019, *p* = .891, *η*_*p*_^*2*^ < 0.001, showed that there were no significant differences in anticipated infantilising behaviours between participants who received a diagnosed patient compared to an undiagnosed patient. Additionally, there was no significant interaction, *F*(1,238) = 1.01, *p* = .316, *η*_*p*_^*2*^ = 0.004, revealing that participants anticipated that they would use more infantilising behaviour toward older adults, independent of the patient's diagnosis.

## DISCUSSION

This study aimed to examine whether patient age and the presence of a BPD diagnostic label influence clinical psychology trainees' treatment beliefs and anticipated reactions to a patient with BPD symptoms. We predicted that clinical psychology trainees' stigmatising judgements would be greatest when behaviours consistent with BPD were described with a BPD label. Contrary to predictions, participants expected worse therapeutic relationships with and showed less conviction in the treatability of, undiagnosed patients. Diagnostic label and age had no effects on cognitive, emotional or distancing responses to the patient. Consistent with expectations, though, participants anticipated that they would use more infantilising behaviours in relation to older patients, irrespective of diagnostic label.

The work by Lam et al. (2015, 2016) critically established the case for the BPD diagnostic label, rather than the behaviour it denotes, activating negative treatment beliefs about this patient group. However, in the present study, the BPD label mitigated the negative treatment beliefs elicited by symptoms of the disorder alone, especially for younger patients. This finding is consistent with more recent research indicating that public feelings of anger, blame and fear were greatest when behaviours consistent with BPD were described without a diagnostic label (Amestoy et al., 2024; Masland & Null, 2022). The absence of a diagnosis might elicit uncertainty surrounding what to expect from the patient's ability to interact with others and how to treat an unknown disorder. Thus, the present findings might reflect a recent reduction in the stigma associated with the BPD label among psychology trainees specifically, although previous research suggests that nurses' attitudes toward BPD have also improved (Day et al., 2018).

The present study was conducted after the British Consensus Statement on Personality Disorder, a policy position that reframed how services understand and respond to personality disorder. Specifically, it marked a shift from viewing personality disorders as untreatable and exclusionary (Lamb et al., 2018) and informed nationwide attempts to reform personality disorder care within UK mental health systems (Morgan et al., 2022). It is conceivable, then, that through these attempts to raise awareness of BPD stigma and improve training in evidence‐based treatments, this diagnosis no longer elicits perceptions that patients are untreatable or unable to form positive therapeutic relationships. Trainees might be exposed to such initiatives through clinical placements and the increasing involvement of people with lived mental health experience within teaching (Kerry et al., 2023).

Previous research suggests that BPD is often overlooked in older adults (Beatson et al., 2016), while training in ageing varies considerably across psychology programmes (Graham & Moye, 2023). Indeed, only participants assigned to a younger patient made significantly more positive predictions about the therapeutic alliance for the diagnosed compared with the undiagnosed patient in the present study, which may reflect a greater emphasis on working with younger adults during training (see McMain et al., 2015). As such, trainees may feel prepared to interact with younger patients with a BPD diagnosis, while experiencing similar levels of uncertainty surrounding therapeutic relationships with diagnosed and undiagnosed older patients with BPD. This may suggest benefit in familiarising trainees with BPD symptoms—particularly in older adults, whose presentations may be less familiar—rather than withholding or relabelling the diagnosis (e.g., Kulkarni, 2017; Sisti et al., 2016).

Arguing that a BPD diagnosis may be beneficial immediately raises the question: ‘Beneficial to whom?’ Ideally, of course, one might hope that a diagnosis would be useful to the patient in fostering insight and aiding treatment planning (Hayne, 2003; Loughland et al., 2015; Pitt et al., 2009; Tedesco et al., 2023). Sulzer et al. (2016) conveyed that most patients with BPD preferred to be informed of their diagnosis, with those who found it had been withheld consistently terminating treatment, suggesting that disclosure can be important for positive therapeutic relationships.

Nevertheless, one must also consider that receiving this diagnosis may elicit negative thoughts about the self (Quenneville et al., 2020; Rüsch et al., 2006; Scheel et al., 2014). For example, in a sample of ten White British personality disorder patients, some interpreted the diagnosis as indicating that there was something fundamentally “wrong” with them as a person (Stalker et al., 2005). Although more diverse patient perspectives on the BPD diagnosis require investigation, such beliefs can result in higher illness severity and poor care adherence (Quenneville et al., 2020). Therefore, the present study emphasizes that any definitive decision to disclose this diagnosis should be guided by a careful consideration of all factors, including patient preference and not just concerns around clinicians' responses.

The findings of this study cannot speak to how the BPD diagnostic label or age may influence qualified practitioners, nor do they permit differentiation between those at more advanced stages of training. As has previously been demonstrated with nursing and psychiatry students, attitudes may become more negative with greater exposure to clinical practice toward the end of training or upon qualification (Lindell‐Innes et al., 2023; Lugboso & Aubeeluck, 2017). The bias of crowds model (Payne et al., 2017) predicts that stigma is a direct reflection of the environment in which we are situated. Therefore, ageism or BPD stigma might be unintentionally transmitted via role‐modelling in healthcare settings where such attitudes may persist (Klein et al., 2022) and could reasonably be expected to later shape clinical psychology trainees' attitudes.

As contemporary UK clinical psychology training programmes typically endorse formulation in preference to diagnosis (Carey & Pilgrim, 2010; Johnstone, 2017), one may also question whether it is more pertinent to explore how diagnosis‐driven professions perceive the BPD label. Although perceptions of this diagnosis are often more negatively entrenched for nurses and psychiatrists than clinical psychologists (Bodner et al., 2015), the present findings nonetheless indicate that the BPD label still influences clinical psychology trainees' judgements—albeit in a more positive manner than traditionally assumed. This may speak to a broader dilemma faced by trainee clinical psychologists: They are taught to conceptualize patients' difficulties without relying on psychiatric labels, yet they operate within healthcare contexts where distress is frequently considered through a diagnostic lens. Hence, although research is required to explore the impact of a BPD diagnosis across disciplines and levels of experience, the present findings offer cautious optimism: The burgeoning population of patients with a BPD diagnosis—old and young—may receive improved care from the next generation of clinical psychologists and CAPs.

According to the Communication Predicament of Ageing Model (CPAM), anticipated infantilising behaviours are activated by stereotypes portraying older adults as senile, incompetent or dependent (Lederman & Shefler, 2022; Shaw & Gordon, 2021). This poses the question: Why did trainees endorse infantilising behaviour toward older patients if they did not also endorse old age stereotypes? Several studies demonstrate that people often keep biases, including age stereotypes, out of awareness (Chopik & Giasson, 2017; Goedderz & Hahn, 2022). Clinicians claim that infantilising behaviours like louder speech or simple vocabulary accommodate older adults' hearing impairments or organic illness (Gould et al., 2002; Shaw & Gordon, 2021). However, the current sample reported intentions to infantilize older patients in the absence of such impairments, suggesting trainees may misattribute stigma to these common rationalisations. Hence, although conclusions regarding how these attitudes might translate into practice should be drawn with caution, ambiguous therapeutic situations may not require trainees to attend to the stereotypes shaping infantilising behaviours.

This indicates that clinical psychology trainees do not endorse stigma toward old age or BPD labels—at least not consciously. As the current study did not include measures of automatic stigma, it cannot ascertain whether participants instead held automatic biases outside of their conscious awareness. We hope that future research will consider incorporating observational methods to detect automatic stigma and actual behaviours toward old age and the BPD diagnosis. Such research may not only provide a more accurate estimate of trainees' negative reactions to older adults with BPD but also point toward effective interventions: encouraging trainees to attend to their stigmatising reactions to old age and BPD in reflective practice (Hahn & Gawronski, 2019).

## CONCLUSION

Clinicians and researchers have consistently asserted that the BPD label and old age are associated with disproportionate stigma when examined in isolation. When age and diagnostic label were considered jointly, trainees anticipated significantly poorer therapeutic alliances for undiagnosed younger adults presenting with BPD symptoms, compared with their diagnosed counterparts. Although trainees reported a greater likelihood of responding to older adults in an infantilising manner, they expressed less conviction in the treatability of undiagnosed patients, irrespective of age. Future research should examine generalisability across professions and experience levels, the role of automatic biases and how these attitudes translate into behaviour.

## AUTHOR CONTRIBUTIONS

**Katie E. Hastings:** Conceptualization; investigation; writing – original draft; methodology; formal analysis; data curation; resources; visualization; writing – review and editing. **Adam Hahn:** Methodology; writing – review and editing; formal analysis; supervision.

## CONFLICT OF INTEREST STATEMENT

The authors declare no conflicts of interest.

## Data Availability

The data that support the findings of this study are openly available in Open Science Framework at https://osf.io/mf3uh/overview?view_only=526ab0f34bcf401e8a47d18d69e6eacc.

## Footnotes

^1^
We preregistered a series of possible result patterns one may expect from previous research but did not repeat them all here due to word count limitations.

^2^

*η*_*p*_^*2*^ refers to partial eta squared, an effect size measure used in ANOVA denoting the proportional reduction in error achieved in a model by inclusion of a specific parameter. It is generally interpreted as small (0.01), medium (0.06) or large (0.14).
